# Endoscopic Resection of Early Gastric Cancer and Pre-Malignant Gastric Lesions

**DOI:** 10.3390/cancers15123084

**Published:** 2023-06-07

**Authors:** Ana Clara Vasconcelos, Mário Dinis-Ribeiro, Diogo Libânio

**Affiliations:** 1 Department of Gastroenterology, Porto Comprehensive Cancer Center Raquel Seruca, and RISE@CI-IPO (Health Research Network), 4200-072 Porto, Portugal; 2 MEDCIDS (Department of Community Medicine, Health Information, and Decision), Faculty of Medicine, University of Porto, 4200-319 Porto, Portugal

**Keywords:** gastric cancer, endoscopy, treatment, endoscopic submucosal dissection

## Abstract

**Simple Summary:**

Although its incidence and the mortality with which it is related seem to be decreasing, gastric cancer remains the fifth most common cause of new cancer cases and the fourth most lethal cancer worldwide. Late diagnosis occurs in a substantial portion of patients, but the increased identification of risk factors and precancerous conditions has allowed for the stratification of risk, leading to tailored patient surveillance and the early recognition of pre-malignant and malignant lesions. Since the 1990s, innovative endoscopic resection techniques have revolutionized the treatment of early gastric cancer, which would otherwise be subject to surgical resection.

**Abstract:**

Early gastric cancer comprises gastric malignancies that are confined to the mucosa or submucosa, irrespective of lymph node metastasis. Endoscopic resection is currently pivotal for the management of such early lesions, and it is the recommended treatment for tumors presenting a very low risk of lymph node metastasis. In general, these lesions consist of two groups of differentiated mucosal adenocarcinomas: non-ulcerated lesions (regardless of their size) and small ulcerated lesions. Endoscopic submucosal dissection is the technique of choice in most cases. This procedure has high rates of complete histological resection while maintaining gastric anatomy and its functions, resulting in fewer adverse events than surgery and having a lesser impact on patient-reported quality of life. Nonetheless, approximately 20% of resected lesions do not fulfill curative criteria and demand further treatment, highlighting the importance of patient selection. Additionally, the preservation of the stomach results in a moderate risk of metachronous lesions, which underlines the need for surveillance. We review the current evidence regarding the endoscopic treatment of early gastric cancer, including the short-and long-term results and management after resection.

## 1. Introduction

Gastric cancer (GC) remains an important cause of cancer worldwide, ranking fifth in new cancer cases and fourth in terms of mortality [1], although incidence and mortality rates have been decreasing in recent decades [2]. Nonetheless, GC was still responsible for just over 1 million new cases in 2020 [1], which is predicted to increase to 1.8 million worldwide by 2040 [3]. A recent study [4] projecting cancer incidence between 2015 and 2050 in the United States of America estimates not only an increase in the absolute number of new GC cases (explained by an aging population) but also a 7% increase in age-standardized incidence rates from 7.5 to 8.0 per 100,000. These numbers underline the importance of healthcare systems’ adaptability to an increasing burden of disease, shifting focus to primary prevention and early detection.

The knowledge of gastric carcinogenesis (namely, the Correa cascade [5]) and the subsequent recognition of gastric premalignant conditions and lesions, the current widespread use of esophagogastroduodenoscopy, and the implementation of national screening programs in high-risk countries such as Japan and South Korea [1,6,7] are expected to result in an increase in the diagnosis of GC at earlier stages.

Early gastric cancer (EGC) comprises gastric malignancies that are confined to the mucosa or submucosa, irrespective of the status of lymph node metastasis (LNM) [8]. The presence of LNM constitutes one of the most relevant prognostic factors among patients with GC, including EGC, which is associated with significantly lower long-term survival [9]. While the standard curative treatment of GC had once been gastrectomy with lymphadenectomy, the development of advanced endoscopic resection techniques has surpassed surgery as a first-line curative treatment for selected early lesions presenting a minimal risk of LNM. However, up to 20% of endoscopic resections do not meet curative criteria and require further surgical treatment [10,11,12], highlighting the need to improve clinical staging and patient selection.

This review aims to provide a comprehensive overview of the endoscopic management of EGC, the challenges physicians still face in their daily practices, and the technical and technological advances designed to overcome these difficulties. 

## 2. Superficial Gastric Lesions

Superficial gastric lesions are made up of premalignant neoplastic lesions and malignant lesions that do not invade beyond the submucosa [13]. The Vienna classification provides a consensus terminology of epithelial neoplasia of the gastrointestinal tract [14]. In the stomach, low-grade dysplasia, high-grade dysplasia, and carcinoma in situ (group 3 and subgroups 4-1 and 4-2 of the Vienna classification, respectively) are considered premalignant lesions in that they are confined to the epithelial layer and do not invariably progress to invasive carcinoma. Invasion into the *lamina propria* or the *muscularis mucosae* constitutes intramucosal carcinoma (subgroups 4-3 and 4-4 of the Vienna classification), which, in the stomach, is considered a malignant lesion, contrary to what is seen in the colon. Additionally, a carcinoma that invades the submucosa (group 5 of the Vienna classification) is also considered a superficial gastric lesion.

The Paris classification, a morphological classification developed in 2003 and updated in 2005, categorizes superficial neoplastic lesions of the gastrointestinal tract into three groups [13]. Type 0-I includes protruding superficial lesions, also known as polypoid lesions, and is subdivided into pedunculated (0-Ip) and sessile (0-Is) lesions. Type 0-II encompasses non-protruding non-excavating lesions, otherwise known as flat lesions, and is made up of slightly elevated (0-IIa), completely flat (0-IIb), and slightly depressed (0-IIc) lesions. It is common for mixed lesions to occur, containing concomitant depressed and elevated components, and such lesions are classified as type “0-IIa + IIc” or “0-IIc + IIa” depending on the predominant component. Finally, type 0-III lesions are excavated (or ulcerated) and can also be mixed with depressed (0-IIc) lesions. This endoscopic classification seems to correlate with histological findings and resection outcomes since a depressed morphology is associated with submucosal invasion and excavated lesions are associated with piecemeal resection. Although subject to interobserver variability, this classification’s reliability is acceptable and improves both with training and the use of virtual chromoendoscopy [15].

## 3. Indications for Endoscopic Resection: Pre-Procedural Evaluation

The reported rate of LNM in intramucosal adenocarcinomas varies between 0% and 9% and can reach up to 25% in adenocarcinomas with submucosal invasion [16,17,18,19]. In certain circumstances, this risk is minimal or even null. The studies conducted by Gotoda et al. [16], Nakahara et al. [17], and Hirasawa et al. [18] evaluated the incidence of LNM in gastrectomy specimens, analyzing the endoscopic and histological characteristics associated with a very low risk of LNM in cases of EGC. These studies served as the cornerstone for the definition of the current criteria for endoscopic resection. More recently, the findings of Hasuike et al. [20] and Takizawa et al. [21] contributed to the expansion of indications for endoscopic resection.

The Japanese and European guidelines recommend endoscopic resection as the standard treatment for gastric lesions harboring dysplasia and for EGC when the presumed risk of lymph node metastasis is less than 1% [22,23]. The Japanese guidelines define expanded indication lesions as lesions that are presumed to have a <1% risk of LNM but for which long-term outcomes were not confirmed by a prospective confirmatory trial with 5-year survival as the primary endpoint [22]. The European guidelines state that EGC with an LNM risk presumed to be inferior to 3% can be considered for endoscopic resection as an expanded criterion, although the decision should consider the patient’s characteristics and preference after the discussion of risks [23]. 

The absolute criteria for endoscopic resection, according to the Japanese guidelines, are gastric lesions clinically staged as (i) dysplastic regardless of size, (ii) differentiated gastric intramucosal (cT1a) adenocarcinomas of any size if not ulcerated and ≤30 mm in size if ulcerated, and (iii) poorly differentiated gastric intramucosal (cT1a) adenocarcinomas without ulcerative findings and ≤20 mm in size [22,24]. The European guidelines, on the other hand, consider the first two groups of lesions as absolute indications for endoscopic resection and the third one as an expanded indication [23]. In these cases, the decision to pursue endoscopic treatment should be individualized following the discussion of the potential risks and benefits of the different treatment options with the patient. The Japanese guidelines define lesions as expanded indications when a previously resected lesion meeting the endoscopic curability criterion eCura C-1 (see Section 6) locally recurs as a clinically staged intramucosal (cT1a) cancer [22,24] (Table 1).

Although endoscopic resection is considered to result in high rates of curative resection, approximately 15–20% of the resected lesions do not meet curative criteria [10,11,12]. Several authors have sought to establish predictive factors for non-curative resection in order to improve patient selection. A 2019 systematic review and meta-analysis identified location in the upper third of the stomach (odds ratio (OR) 1.49, 95%CI 1.24–1.79), depressed morphology (OR 1.49, 95%CI 1.04–2.12), and lesions whose identified characteristics lie outside standard criteria (OR 3.56, 95%CI 2.31–5.48) as predictors of this outcome [25]. Additional risk factors identified in individual studies include large tumor size (generally >20 mm), ulceration, undifferentiated tumors (including the presence of an undifferentiated component in differentiated-type-predominant mixed-type lesions), and old age [26,27,28,29,30,31]. Regarding lesion differentiation, a meta-analysis incorporating 5644 patients showed that undifferentiated-predominant mixed-type lesions show more aggressive biological behavior compared to pure undifferentiated-type lesions, presenting a significantly higher risk of submucosal invasion (OR 2.19, 95%CI 1.90–2.52) and LNM (OR 2.28, 95%CI 1.72–3.03) even after stratification for depth of tumor invasion [32]. 

Furthermore, deep submucosal invasion (>500 µm, ≥Sm2) is an independent risk factor for LNM and a major criterion of non-curability [16,17,18,23,33,34,35]. Thus, accurately estimating the depth of invasion is one of the most important components of an endoscopic preoperative assessment but also one of the most challenging. A few authors have attempted to identify macroscopic features suggestive of Sm2 invasion. Abe et al. [36] suggested that remarkable redness, an uneven surface, margin elevation, enlarged folds, a tumor size >30 mm, and ulceration were significantly associated with deeper submucosal invasion.

Magnifying endoscopy, usually applied in combination with narrow-band imaging, is an ancillary tool for the diagnosis of EGC. Several authors have evaluated whether certain vascular and surface patterns could predict the histologic type and depth of invasion of a tumor; however, there is not yet a gastric classification comparable to the ones of colonic polyps and esophageal lesions. Nakayoshi et al. [37] and Yokoyama et al. [38] found that a fine network microvascular pattern was associated with differentiated lesions, while a corkscrew pattern was associated with undifferentiated histology. What Nakayoshi et al. considered to be an unclassified pattern was designated as an intra-lobular loop pattern by Yokoyama et al., which subdivided it into type 1 (predictive of differentiated-type EGC) and type 2 (found in both differentiated and undifferentiated lesions). Tanaka et al. [39] found that a microsurface pattern of irregular arrangements and sizes was the predominant type in differentiated tubular adenocarcinomas (although depressed adenomas also presented the same pattern), while all signet-ring cell carcinomas and poorly differentiated tubular adenocarcinomas showed a destructive microsurface pattern. Ok et al. [40] concluded that the magnification patterns with narrow-band imaging could aid in predicting histopathology; specifically, a fine network or loop microvascular pattern was associated with differentiated tumors, while an absent microsurface pattern and corkscrew microvascular pattern were associated with undifferentiated tumors. Furthermore, a destructive microsurface pattern was associated with submucosal invasion. Kanesaka et al. [41] found that absent microsurface and opened-loop microvascular patterns did not improve the overall accuracy of white light endoscopy for the diagnosis of undifferentiated-type EGC in depressed or flat lesions, although it improved specificity. 

Different modalities for local staging, the foremost of which is endoscopic ultrasonography, have not proven to be superior to endoscopic evaluation in assessing depth of invasion; consequently, European guidelines do not recommend such modalities’ routine use [23]. Computed tomography and positron emission tomography also have no role in the pre-resection evaluation of endoscopically resectable EGC since the risk of distant metastasis is very low.

**Figure 1:**
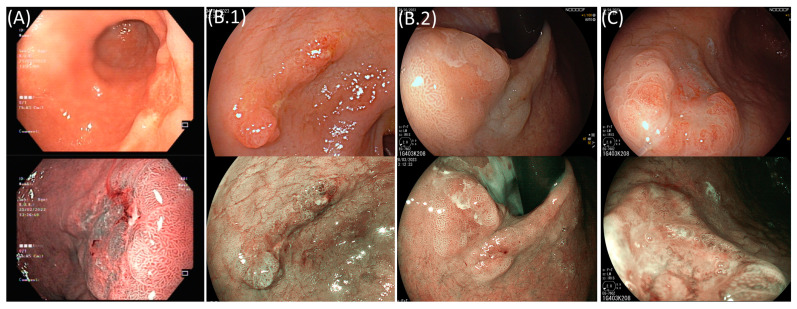
Superficial gastric lesions (upper image—white light; lower image—virtual chromoendoscopy). (**A**) A Paris 0-IIa+Iic lesion clinically staged as deep submucosal invasion in pre-resection endoscopic evaluation (pT1b, undifferentiated, and with lymphovascular invasion on surgical specimen). (**B**) Lesions successfully removed using ESD and meeting curative criteria. (**B.1**) A Paris Iia+Iic 40 mm lesion (pT1a, well-differentiated, and no lymphovascular invasion). (**B.2**) A Paris Iia+Iic 15 mm lesion (pT1a, well-differentiated, and no lymphovascular invasion). (**C**) A Paris 0-Iia+Iic 12 mm lesion that was endoscopically resected and did not meet curative criteria (pT1b, well-differentiated, and with lymphovascular invasion).

Therefore, endoscopic resection should only be proposed to a patient should after a careful evaluation of the gastric lesion by an experienced endoscopist, who should look for endoscopic features associated with non-curability and account for clinical and pathological characteristics (Figure 1).

## 4. Endoscopic Resection

The endoscopic resection of gastric dysplastic lesions and EGC can be carried out by performing endoscopic mucosal resection (EMR) or endoscopic submucosal dissection (ESD).

EMR was first described in 1993 [42] for the endoscopic treatment of esophageal, gastric, and colonic lesions. Before resection, the lesion is elevated through the injection of a solution in the submucosal space to separate it from the *muscularis propria.* The lesion is then placed within a metal wire snare and resected using high-frequency diathermy. This procedure is effective and safe. However, the size of the snare generally prevents the en bloc resection of larger lesions. Piecemeal and/or incomplete resection limits proper histopathological evaluation and staging, which are crucial for post-resection management and associated with local recurrence [43]. ESD was developed in 1995 [44] to overcome the limitations of EMR, allowing for the en bloc resection of lesions of any size. In this method, the lesion is circumferentially outlined with coagulation marks and then elevated after the injection of a solution in the submucosal layer. The endoscopist makes three to four electrosurgical incisions in the coagulation marks to access the submucosa and completes a circumferential incision around the lesion. Finally, the submucosa is dissected in the submucosal plane to achieve an en bloc resection.

Several retrospective and prospective studies and meta-analyses have been carried out to compare the safety and efficacy outcomes between EMR and ESD (Table 2) [45,46,47,48]. ESD is significantly superior to EMR in achieving en bloc and complete resection for lesions of any size, resulting in significantly higher rates of curative resection and lower recurrence. Regarding safety, ESD and EMR present similar levels of post-procedural bleeding, while ESD is associated with higher perforation rates and operative time. Indeed, ESD continues to show high rates of en bloc and complete resection (over 95% and 90%, respectively) and low local recurrence (<5%) and low rates of adverse events, namely, perforation (<3%) and post-operative bleeding (≈5%) [11,49,50]. The endoscopic resection of gastric superficial lesions is associated with a good long-term prognosis, with 5-year overall (OS) and disease-specific survival (DSS) rates of 89.0–95.0% and >99%, respectively [50,51,52].

Accordingly, ESD is the recommended first option for the endoscopic treatment of gastric superficial lesions deemed resectable [22,23]. The European guidelines state, however, that EMR should be considered for elevated lesions (Paris 0-Iia), under 10 mm in size and with a low likelihood of advanced histology.

Endoscopic resection, although safer than gastrectomy, can also present adverse events. Predicting these outcomes can assist in patient selection and help plan periprocedural measures for the prevention of such outcomes. 

Post-procedural bleeding is the most frequent adverse event following ESD, occurring in 4.4–5.1% of procedures [11,53], and it is linked to prolonged hospital stays, the requirement for transfusion, endoscopic reintervention, surgery, and death. A meta-analysis identified risk factors for PPB, which were either patient-, lesion-, or procedure-related [53]. The risk factors associated with this unfavorable outcome were a male gender, cardiopathy, antithrombotic drug use, cirrhosis, chronic kidney disease, a tumor size > 20 mm, a resected specimen >30 mm size, localization in the lesser curvature, a flat or depressed morphology, carcinoma histology, ulceration, a procedure duration of >60 min, and the use of histamine-2 receptor antagonists as an acid-suppressive therapy instead of proton pump inhibitors. The latter reduce the rate of delayed bleeding [54,55], and their administration following ESD is recommended [22]; however, a meta-analysis showed that premedication with proton-pump inhibitors does not impact bleeding rates, despite significantly increasing gastric pH at the time of ESD [56]. Coagulation of visible vessels in post-ESD ulcers is also associated with reduced rates of delayed bleeding [57] and is a recommended preventive measure [22]. A network meta-analysis evaluated additional preventive measures and found that tissue shielding with polyglycolic acid significantly reduced delayed bleeding risk in high-risk patients [risk ratio (RR) 0.32; 95%CI 0.12–0.79], while hemostatic spray potentially reduced bleeding in low-risk patients, although heterogeneity was high [58].

Perforation is an uncommon adverse event of ESD and can be immediate (<3% of procedures) or delayed (<1%). A meta-analysis identified the following as risk factors for perforation: liver disease, location in the upper stomach, a resection size > 20 mm, submucosal invasion, operation time > 2 h, depressed or flat lesions, and piecemeal resection [59]. Another meta-analysis, this time comparing gastric ESD in elderly and non-elderly patients, found a trend for significantly increased perforation risk among patients aged >80 years [60].

## 5. Endoscopic Resection versus Surgery

Resection of the stomach and regional lymph nodes is the standard surgical curative treatment for GC, entailing the removal of at least two thirds of the stomach and a D2 lymph node dissection [24]. This ensures high rates of complete resection, almost negligible rates of local recurrence, a very low risk of metachronous lesions, and high disease-free and overall survival. On the other hand, surgical resection has its own adverse events; it can significantly impact the stomach’s storage and digestive functions, thereby limiting nutrient absorption; and the resulting effects may impair the patient’s health-related quality of life.

Alternatively, ESD is a minimally invasive procedure that preserves the stomach’s structure and associated functions and presents a low rate of complications and adverse outcomes. The spared mucosa constitutes, however, a sustained risk for metachronous tumors, thereby demanding long-term surveillance.

Several meta-analyses have compared the short (Table 3) and long-term (Table 4) outcomes of ESD versus surgery for the treatment of EGC [61,62,63,64,65]. Endoscopic treatment is associated with significantly decreased operation times, in-hospital stays, and overall postoperative complication rates, with one meta-analysis also reporting a lower risk of procedure-related death (OR 0.21, 95%CI: 0.07–0.68) [64]. On the other hand, the rates of en bloc resection, complete resection, and curative resection seem to be significantly lower for ESD compared to surgery (OR 0.07, 95%CI 0.03–0.21; OR 0.07, 95%CI 0.03–0.14; and OR 0.06, 95%CI 0.01–0.27, respectively) [64], resulting in higher rates of recurrence. However, Gu et al. [62] found that the proportion of patients that were amenable to radical treatment after recurrence was higher in the ESD group incorporated in their study (OR 5.27, 95%CI 2.35–11.79). Synchronous and metachronous cancers have been found to be significantly more prevalent after ESD. Regarding long-term outcomes, the differences in 5-year disease-free survival (DFS) are not homogeneous across studies. Some authors found no statistically significant differences [61,63], while others state a significantly lower DFS in their respective ESD groups [62,64,65]. This may be due to differences in defining disease-free survival. Abdelfatah et al. [61] did not incorporate the detection of metachronous lesions as a disease-defining event, Gu et al. [62] included metachronous GC occurrence in the definition of DFS, and the remaining authors [63,64,65] did not specify which events defined DFS. However, the ESD and surgery groups consistently showed similar 5-year overall and disease-specific survival (OS > 95% and DSS > 99% in both groups) throughout the different meta-analyses.

Gastric cancer with an undifferentiated histology presents a significantly higher risk of lymph node metastasis than differentiated tumors [16,66]. Several comparative studies have been performed to compare long-term outcomes, namely, survival, in patients undergoing ESD and surgery for undifferentiated mucosal tumors with a diameter <20 mm and without ulcerative findings. Two meta-analyses summarizing the evidence collected were recently conducted [67,68]. The results overlap with those stated above for general cohorts, with ESD showing a significantly lower 5-year DFS, no statistical difference in DSS, and similar OS (Table 4).

A number of studies have reported on the hospital costs associated with either procedure [69,70,71]. ESD seems to account for significantly lower costs when compared to surgery, which is mostly due to the nature of the procedure itself and differences in the length of stay. Shin et al. [70] evaluated costs related to general cases, stating that given the superior rate of adverse events following surgical resection, the difference in costs may be higher than estimated. Kim et al. [69] also compared medical costs linked to follow-up at 1-year post-discharge and did not find significant differences.

**Table 3:** Summary of meta-analyses comparing short-term outcomes between endoscopic submucosal dissection and gastrectomy patients.

Author, Year	Type of Resection	Operation Time (in Minutes)	in-Hospital Stay (in Days)	Overall Postoperative Complication	Recurrence	Synchronous Lesions	Metachronous Lesions
Abdelfatah MM, 2019 [61]	ESD	ND	ND	ND	40/2943 (1.4%)	16/1082 (1.5%)	176/2943 (6%)
	Gastrectomy				12/3116 (0.4%)	1/1485 (0.1%)	13/3116 (0.4%)
	-				OR 0.17 (0.1–4.9)	RR 5.7 (1.5–21.9)	RR 10.2 (5.9–17.1)
Gu L, 2019 [62]	-	ND	ND	ND	ND	OR 4.94 (3.04–8.03)	OR 8.64 (5.00–14.95)
Li H, 2020 [63]	-	WMD −140 (−254 to −34)	−5.41 (−5.93 to −4.89)	OR 0.39 (0.28–0.55)	OR 9.24 (5.94–14.36)	ND	ND
Liu Q, 2020 [64]	-	MD −128 (−204 to −52)	−7.13 (−7.98 to −6.28)	OR 0.47 (0.34–0.63)	OR 5.42 (2.91–10.11)	OR 6.59 (1.96–22.1)	OR 10.84 (6.43–18.26)
Xu X, 2022 ^a^ [65]	-	ND	ND	OR 0.49 (0.34–0.72)	ND	OR 9.09 (2.17–50)	OR 8.33 (4–20)

MD: mean difference; ND: no data; OR: odds ratio; WMD: weighted mean difference. ^a^ Expanded indication lesions. The 95% confidence intervals are shown in parenthesis.

**Table 4:** Summary of systematic reviews with meta-analyses comparing long-term survival between endoscopic submucosal dissection and gastrectomy patients.

Author, Year	Type of Resection	Overall Survival	Disease-Specific Survival	Disease-Free Survival
Abdelfatah MM, 2019 [61]	ESD	2914/3034 (96%)	2437/2451 (99.4%)	1415/1476 (95.9%)
	Gastrectomy	3088/3203 (96%)	1962/1977 (99.2%)	1816/1844 (98.5%)
	-	OR 0.96 (0.74–1.25)	OR 0.7 (0.16–2.9)	OR 1.86 (0.57–6.0)
Gu L, 2019 [62]	ESD	2238/2324 (96.3%)	5/1425 (99.7%)	1241/1376 (90.2%)
	Gastrectomy	2563/2662 (96.3%)	17/1841 (99.1%)	1261/1298 (97.2%)
	-	RR 0.90 (0.68–1.19)	RR 0.40 (0.15–1.03)	RR 3.40 (2.39–4.84)
Li H, 2020 [63]	-	HR 0.51 (0.26–1.00)	ND	ND
Liu Q, 2020 [64]	-	HR 0.92 (0.71–1.19)	HR 0.73 (0.36–1.49)	HR 4.58 (2.79–7.52)
Huh CW, 2021 ^a^ [67]	-	OR 2.29 (0.98–5.36)	ND	ND
Xu X, 2022 ^b^ [65]	-	HR 1.22 (0.66–2.25)	ND	HR 3.29 (1.60–6.76)
Yang HJ, 2022 ^a^ [68]	ESD	383/400 (95.8%)	396/400 (99.0%)	362/400 (90.5%)
	Gastrectomy	492/508 (96.9%)	506/508 (99.6%)	491/508 (96.7%)
	-	RR 1.18 (0.60–2.32)	RR 2.49 (0.47–37.93)	RR 2.49 (1.42–4.35)

ESD: endoscopic submucosal dissection; HR: hazard ratio; ND: no data; OR: odds ratio; RR: risk ratio. ^a^ Undifferentiated lesions. ^b^ Expanded indication lesions. The 95% confidence intervals are shown in parenthesis for HR, OR, and RR.

Considering that gastrectomy with lymphadenectomy is a major surgical procedure entailing the resection of a considerable portion of the stomach and ESD is a minimally invasive and stomach-sparing procedure, a few authors have evaluated patient-reported quality of life after curative treatment. We found three comparative studies, one of which was retrospective [72] and the other two were prospective [73,74]. In all three, quality of life was assessed using the European Organization for Research and Treatment of Cancer (EORTC) Quality of Life Questionnaire Core 30 (QLQ-C30) and a GC-specific module, namely, the EORTC QLQ-STO22. Song et al. [72] reported a significantly higher overall health status in the ESD group compared to the surgery group (*p* < 0.05) and a global trend in all function and symptom scales in favor of endoscopic treatment, although statistical differences were only found in relation to physical function, social function, fatigue, nausea and vomiting, appetite loss and constipation, reflux, eating restrictions, and body image. Libânio et al. [73] found, at 1-year, a net benefit in overall health favoring ESD (*p* = 0.006). ESD was not associated with worsening in any functional dimensions or symptom scales compared to baseline. This result contrasts with those regarding the surgery group, whose patients reported a significant decrease in role function and worsened fatigue, pain, appetite loss, diarrhea, dysphagia, eating restrictions, taste, and body image. ESD patients did not more frequently report fear of recurrence, new tumors, or death when compared with surgical patients. Kim et al. [74] reported significant differences between groups only with regard to physical functioning, eating restrictions, dysphagia, diarrhea, and body image.

Taking all the above into account, when a superficial gastric lesion is amenable to endoscopic resection with a high likelihood of curability, guidelines consider endoscopic resection to be a more desirable choice of curative treatment compared to surgery [23]. Especially in cases of expanded indications, this should be a shared decision between a patient and their physician that is finalized after a discussion of the advantages and downsides of both treatment modalities with respect to both short- and long-term outcomes [75].

## 6. Management after Resection

After endoscopic resection, a pathological examination is essential in order to properly characterize the resected lesion and classify the resection as curative or non-curative, thereby guiding posterior management. The criteria for curability regarding resections have been defined according to the risk of LNM based on the histological findings of surgical specimens. Several studies throughout the years have consistently identified lymphovascular invasion, deep submucosal invasion (>500 µm), undifferentiated histology, and a size ≥30 mm as independent risk factors for LNM [16,17,18,23,33,34,35], and this evidence is the cornerstone for the definition of current curative criteria.

**Figure 2:**
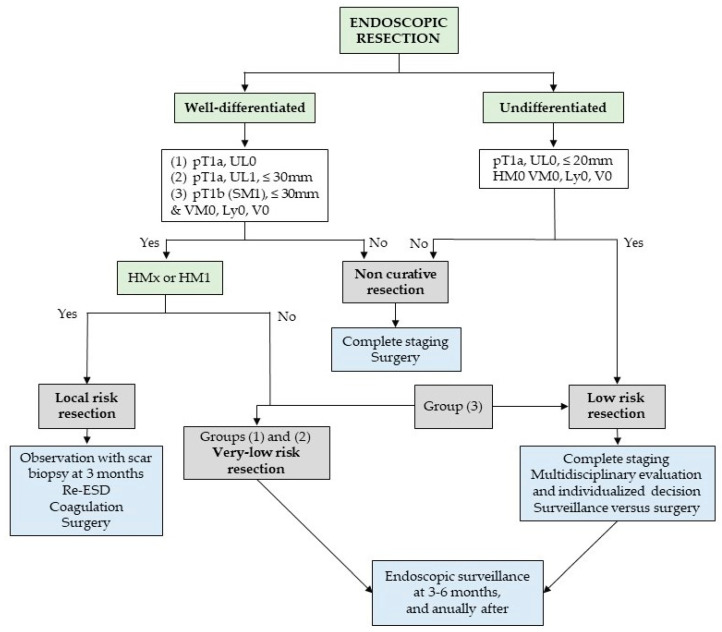
Post-resection management according to the European Society of Gastrointestinal Endoscopy guidelines on ESD. pT1a: intramucosal adenocarcinoma. pT1b (SM1): adenocarcinoma with superficial submucosal invasion (≤500 µm). UL0: non-ulcerated. UL1: ulcerated. VM0: negative vertical margin. Ly0, V0: no lymphovascular invasion. HM0: negative horizontal margin. HMx: piecemeal resection. HM1: positive horizontal margin.

European guidelines [23] consider two groups of curative resections (Figure 2):

- Very-low-risk resections (LNM risk < 0.5–1%), i.e., when a differentiated mucosal (pT1a) lesion, without lymphovascular invasion, and independent of size if there are no ulceration findings or ≤30 mm in size if ulcerated, is resected en bloc and with negative margins;
- Low-risk resections (LNM risk <3%), i.e., when a poorly differentiated pT1a lesion ≤ 20 mm in size or a differentiated pT1b lesion (submucosal invasion ≤ 500 µm) ≤30 mm in size, that present neither ulceration nor lymphovascular invasion, is resected en bloc with negative margins.

A very-low-risk resection does not require any further radiological staging or treatment, whereas for lesions meeting low-risk criteria, further treatment is generally not recommended, but the patient should undergo complete staging, and the decision to pursue additional surgical therapy should be individualized after discussion with a multidisciplinary team.

A third group of lesions is classified as local-risk resections—due to a very low risk of LNM but an increased risk of local recurrence—when a piecemeal resection or tumor-positive horizontal margin occurs in (i) lesions otherwise meeting very low risk criteria and (ii) differentiated pT1b lesions with submucosal invasion ≤ 500 µm, a size ≤ 30 mm, and negative vertical margins, provided that there is no evidence of submucosal invasion at the resection margin. Management in such situations should be tailored, for which patient preferences should be considered, with guidelines preferring either close observation with scar biopsy or re-ESD/scar ablation over surgery given its poorer safety profile. However, surgery is an adequate alternative, especially for cases of recurrence that are not amenable to endoscopic re-intervention.

Finally, endoscopic resections are classified as noncurative for any lesion with: positive vertical margins; lymphovascular invasion; deep submucosal invasion (>500 µm from the *muscularis mucosae*); ulceration or a size > 20 mm in poorly differentiated lesions; a size > 30 mm in pT1b differentiated lesions with submucosal invasion ≤500 µm and in intramucosal ulcerated lesions. In these cases, complete staging is recommended, and a further curative resection should generally be pursued, namely, gastrectomy and lymphadenectomy, since the presence of LNM is linked to a poor prognosis. For patients who refuse salvage surgery or are unfit for a major surgical procedure, surveillance may be an acceptable alternative.

The Japanese guidelines [22,24], on the other hand, use the eCura grading system to categorize the curability of resected lesions. Lesions are classified as endoscopic curability A (eCuraA) when the effect of endoscopic resection is equal to or superior to surgery with respect to long-term outcomes. These include the same resections classified as very low risk in European guidelines as well as the en bloc resection of intramucosal (pT1a) predominantly undifferentiated-type lesions that are ≤20 mm and non-ulcerated, possess negative horizontal and vertical margins, and do not present lymphovascular invasion. However, predominantly differentiated lesions with an undifferentiated component > 20 mm are considered non-curative resections (endoscopic curability C-2). When curability can be expected, although there is not yet sufficient evidence of long-term results, lesions are graded as endoscopic curability B (eCuraB), and are constituted by en bloc resection of predominantly differentiated-type lesions with a minute degree of submucosal invasion (≤500 µm from the *muscularis mucosae*, pT1b1), negative horizontal and vertical margins, and no lymphovascular invasion. If an undifferentiated component is present in the submucosal portion of the lesion, the resection is considered non-curative. Every other lesion not fulfilling eCuraA or eCuraB criteria is a non-curative resection and classified as endoscopic curability C lesions (eCuraC). This group subdivides into eCuraC-1, which encompasses differentiated eCuraA or eCuraB lesions that were either not resected en bloc or had positive horizontal margins, and eCuraC-2, which is made up of all other non-curative resections.

Regarding non-curative resections, Libânio et al. [76] reported that 75% of the gastrectomy specimens of such cases did not show residual lesions, and the 5-year DSS did not seem to differ between patients in the surgical and non-surgical groups [76,77]. Thus, indiscriminately recommending surgical treatment to all non-curative resections may be excessive. Accordingly, Hatta et al. [78] created a scoring system for non-curative resections, attributing the following points to five different risk factors for LNM: three points for lymphatic invasion, and one point each for tumors > 30 mm, presenting positive vertical margins, presenting venous invasion, and whose level of submucosal invasion is >500 µm. Patients were then stratified into three groups corresponding to LNM risk: low (zero points to one point: 2.5% risk), intermediate (two to four points: 6.7% risk), and high (five to seven points: 22.7% risk). A validation arm verified that this categorization is associated with significantly different DSS between risk groups (99.6, 96.0, and 90.1% at 5 years, respectively; *p* < 0.001) and that the low-risk group presents very high DSS, which is comparable to that of EGC patients who fulfill curative criteria after endoscopic resection. This tool may be helpful in attempting to predict which patients will receive the most benefit from salvage surgery after non-curative ESD and for whom surgical treatment may represent a riskier option than surveillance.

As stated before, endoscopic resection preserves the stomach at the expense of maintaining gastric mucosa at risk for metachronous lesions and recurrence. The rate of metachronous lesions after curative endoscopic resection described in the literature varies between 3% and 20%. In a recent meta-analysis, Ortigão et al. [79] determined a value of metachronous gastric lesion cumulative incidence at 5 years of 9.5% after endoscopic resection, which was significantly higher than that of 0.7% for surgery, with the meta-regression model predicting an increase in the metachronous rate with time, namely, up to 14.9% at 10 years for endoscopic resection versus 2.3% for surgery. This highlights the need for endoscopic surveillance post-resection.

European guidelines [23] recommend a follow-up endoscopy 3–6 months after a curative resection or local-risk resection without local recurrence and annually thereafter, while Japanese guidelines [24] recommend annual endoscopy for an eCuraA resection and annual or biannual endoscopic surveillance for an eCuraB resection. There are no studies comparing annual and biannual surveillance, but an endoscopy interval less than 12 months does not seem to increase the proportion of metachronous lesions amenable to endoscopic resection [79]. On the other hand, one study found that a surveillance interval greater than 12 months was significantly linked to the recurrence of adenocarcinoma, larger lesions, and a higher proportion of patients undergoing surgical treatment [80].

Still regarding the surveillance interval, multiple studies have tried to find risk factors for metachronous GC to enable the tailoring of surveillance according to individual risk. The aforementioned meta-analysis found the following to be significantly associated with metachronous: older age (mean difference 1.08 years, 95%CI 0.21–1.96), male sex (OR 1.43, 95%CI 1.22–1.66), a family history of GC (OR 1.88, 95%CI 1.03–3.41), synchronous lesions (OR 1.72, 95%CI 1.30–2.28), severe gastric mucosal atrophy (OR 2.77, 95%CI 1.22–6.29), intestinal metaplasia in corpus (OR 3.15, 95%CI 1.67–5.96), a persistent Helicobacter pylori infection (OR 2.08, 95%CI 1.60–2.72), and a lower pepsinogen I/II ratio (mean difference–0.54, 95%CI −0.86 to −0.22) [79]. 

Several meta-analyses have evaluated the impact of *H. pylori* eradication on the risk of metachronous lesions following an endoscopic resection of EGC and generally concluded that eradication is associated with reduced rates of metachronous GC [RR 0.46, 95%CI 0.37–0.57 [81]; RR 0.467, 95%CI 0.362–0.602 [82]; RR 0.50, 95%CI 0.41–0.61 [83]; OR 0.42, 95%CI 0.32–0.56 [84]; OR 0.47, 95%CI 0.33–0.67 [85]; hazard ratio (HR) 0.43, 95%CI 0.26–0.70 [86]. One meta-analysis [85] incorporating 6967 patients from nine randomized controlled trials found that there was no difference in metachronous incidence when patients had already-established atrophic gastritis and intestinal metaplasia at baseline. International guidelines [22,24,87] recommend that a patient’s *H. pylori* status be determined after the endoscopic resection of EGC, with reflex eradication.

Finally, the required duration of a follow-up after resection has not not clearly defined, and neither is the level of expertise of the endoscopists assigned to this task. The risk of metachronous lesions is higher for older patients but also seems to increase with time for up to 10 years after resection (even among younger patients). In one study, a survival analysis showed a stable cumulative incidence of metachronous cancer 10 years post-resection [88].

## 7. Future Perspectives

Predicting the depth of invasion of EGC is one of the most challenging aspects of the endoscopic assessment of superficial gastric lesions. Artificial intelligence (AI) systems have been used in several medical fields. A few studies have undertaken the evaluation of the accuracy of AI systems in predicting the depth of invasion of EGC. Zhu et al. [89] and Tang et al. [90] report an accuracy of around 88–89% for predicting tumor depth, while Yoon et al. [91] report a sensitivity and specificity of 79.2% and 77.8%, respectively. Nagao et al. [92] evaluated an AI system’s ability to predict depth of invasion using conventional white-light imaging, non-magnifying narrow-band imaging, and indigo-carmine dye contrast imaging and found no differences, with accuracies varying between 94.5% and 95.5%. Wu et al. [93] report a lower accuracy of 78.57% for predicting EGC invasion depth, which is still comparable to endoscopists’ results, and Hamada et al. [94] present similar accuracy values (78.9–82.4%, depending on whether evaluations were image-based or lesion-based). Two systematic reviews with meta-analyses have assessed the performance of AI systems with respect to estimating depth invasion [95,96]. The pooled sensitivity and specificity for predicting deep submucosal invasion were 72–82% and 79–90%, respectively. Jiang et al. concluded that AI-assisted depth diagnosis is more accurate than that of experts, while Xie et al. did not find differences on this matter. Kim et al. [97] compared two AI models, one developed from static images and the second from video clips, and concluded that models developed from videos could predict EGC depth invasion more precisely than image-trained models. A recent study [98] suggests that human–machine cooperation improves performance when compared to the individual results of either one. Although promising, AI systems have yet to prove themselves more accurate than experts at predicting depth of invasion. Therefore, they have not been implemented in clinical practice; however, the technology is expected to improve quickly.

There also seems to be room for improvement in cases of non-curative resection, as the search for the less invasive management of GC continues. As mentioned previously, a great portion of lesions that do not meet curative criteria fail to show residual disease or LNM after rescue surgery. Given the post-surgery morbidity and impact on quality of life of gastrectomy, it would be desirable to avoid surgery among patients who have not yet developed LNM. In this regard, Abe et al. [99] first described in 2005 a minimally invasive strategy combining ESD followed by laparoscopic lymph node dissection (LLND). Theoretically, in a patient with a lesion that has been completely resected via ESD but with a clinically significant risk of LNM, LLND would offer the potential to confirm the absence of LNM, hence obviating the need for gastric resection. The same group evaluated the long-term outcomes of combining ESD and LLND in a group of 21 patients whose lesions were completely removed but presented at least one risk factor for LNM [100]. Fourteen patients had undifferentiated-type lesions, eight had deep submucosal invasion, and two had lymphatic invasion. After a median follow-up of 61 months, none showed evidence of metastatic disease, including two patients with positive lymph node metastasis as determined via LLND who refused salvage surgery and were followed for 78–85 months. The authors also evaluated adverse events resulting from the procedure. Gastric lymph node dissection usually implies the division of major feeding arteries and the resection of vagal trunks, which may result in early or delayed gastric ischemia on the one hand and gastritis, perforation or ulcers, and impaired gastric motility on the other. In this study, one patient suffered gastric perforation from early ischemic gastritis, three patients presented a moderate amount of gastric residue following gastroscopy, and two patients complained of postprandial static symptoms such as abdominal distention and belching.

The consequences related to an extended lymph node dissection may be partially curbed by further limiting the number of patients submitted to radical lymphadenectomy. As already conducted for other cancer types, a strategy of lymph node mapping in GC patients has been under study. A lymph node metastasis diagnosis based on the sentinel lymph node biopsy (SLNB) of patients with a significant risk of LNM after ESD could theoretically avoid unnecessary gastrectomy and/or radical lymphadenectomy. Several meta-analyses have evaluated the diagnostic accuracy of SLNB [101,102,103,104,105]. The identification rate of sentinel nodes varied between 93.7–99.0%, and sensitivity varied between 76.9–92.0%. However, the studies were highly heterogenous, with stark differences in the clinical staging of GC patients, tracers used, methods of injection, comparison groups, and the extent of lymphadenectomy. False negative rates of up to nearly 25% seem unreasonable considering the prognosis of GC patients with LNM. Sensitivity seems to be higher in earlier T stages, with a meta-analysis of cT1N0M0 gastric cancer reporting a sensitivity of 92% [104], and a cohort of two randomized controlled trials reporting a pooled sensitivity of 97.7% for pT1 tumors after subgroup analysis [106]. A single-arm study of the long-term oncologic outcomes of SLNB in cT1 gastric cancer cases, incorporating 100 patients and employing a median follow-up period of 47.5 months, showed a 3-year recurrence-free survival rate of 96.0% (95%CI 92.2–100.0%) and an OS of 98.0% (95%CI 95.2–100.0%) [107].

New minimally invasive strategies such as SLNB or LLND after ESD could eventually lead to the expansion of the indications for the endoscopic resection of EGC. However, there are few studies evaluating the combination of LLND with ESD, and SLNB has not yet shown consistent and satisfactory results, with a high heterogeneity of methods among studies.

## 8. Conclusions

ESD is now established as the preferential endoscopic resection technique for gastric superficial lesions (when compared to EMR) and is also preferable to surgery, offering advantages in terms of morbidity and quality of life. ESD is being successfully implemented in western countries, and in the stomach, the corresponding efficacy and safety outcomes are comparable to eastern studies. As ESD is now recommended as a first-line treatment for lesions with a low risk of LNM, three aspects should drive future research:

1. Prediction of and decrease in adverse events: The identification of patients at higher risk of adverse outcomes is important in order to provide patients with more comprehensive information and implement preventive strategies such as defect closure or defect shielding.
2. Better patient selection: Up to 20% of endoscopically resected lesions still do not meet curative criteria, and it is desirable to improve pre-resection endoscopic assessments to avoid unnecessary procedures conducted on patients who would not benefit from them and to better allocate scarce resources. In this regard, AI will probably have a clear role in assisting endoscopists in treatment allocation.
3. The optimization of the management of patients with non-curative resection: The stratification of the risk of LNM, with individualized predictions, should be pursued; this can be achieved through the refinement of existing scoring systems (eCura) and possibly by incorporating additional variables (and possibly molecular features that can help predict this undesirable outcome of LNM). Less invasive alternatives to gastrectomy with lymphadenectomy among patients with non-curative resections should also be pursued, but more studies are needed to clarify the potential role of LLND and SLNB.

The efficacy of a follow-up after resection is also a matter of debate, with sparse evidence backing such intensive and longstanding protocols. We hope that trials evaluating different surveillance protocols according to a patient’s individual risk of developing metachronous lesions will soon be found.

## Figures and Tables

**Table 1:** Absolute and expanded indications according to European and Japanese guidelines.

Type of Lesion		European Guidelines	Japanese Guidelines
Dysplasia, any size		Absolute indication	
Adenocarcinoma	cT1a, well-differentiated, non-ulcerated, any size	Absolute indication	
	cT1a, well-differentiated, ulcerated, ≤30 mm	Absolute indication	
	cT1a, poorly differentiated, non-ulcerated, ≤20 mm	Expanded indication	Absolute indication
	Recurrence of an eCura-C1 lesion, staged as cT1a	-	Expanded indication

cT1a: adenocarcinoma clinically staged as intramucosal.

**Table 2:** Summary of meta-analyses comparing short- and long-term outcomes between endoscopic mucosal resection and endoscopic submucosal dissection.

Author, Year	Type of Resection	Operation Time (in Minutes)	Perforation Rate	Local Recurrence	En Bloc Resection	Complete Resection
Tao M, 2019 [45]	-	SMD 1.12 (0.13–2.10)	OR 2.55 (1.48–4.39)	OR 0.18 (0.09–0.34)	OR 9.00 (6.66–12.17)	OR 8.43 (5.04–14.09)
Lian J, 2012 [46]	EMR	ND	17/1973 (0.9%)	126/1973 (6.4%)	1020/1973 (51.7%)	867/2053 (42.2%)
	ESD	ND	62/1438 (4.3%)	11/1438 (0.8%)	1328/1437 (92.4%)	1227/1495 (82.1%)
	-	WMD 59.4 (16.8–102.0)	OR 4.67 (2.77–7.87)	OR 0.10 (0.06–0.18)	OR 9.69 (7.74–12.13)	OR 5.66 (2.92–10.96)
Facciorusso A, 2014 [47]	EMR	ND	17/1973 (0.9%)	141/2332 (6.0%)	1020/1973 (51.7%)	867/2053 (42.2%)
	ESD	ND	62/1438 (4.3%)	12/1859 (0.6%)	1328/1437 (92.4%)	1227/1495 (82.1%)
	-	SMD 1.73 (0.52–2.95)	OR 4.67 (2.77–7.87)	OR 0.09 (0.05–0.17)	OR 9.69 (7.74–12.13)	OR 5.66 (2.92–10.96)
Zhao Y, 2018 [48]	EMR	-	26/2134 (1.2%)	116/2245 (5.2%)	1422/2551 (55.7%)	1110/1935 (57.4%)
	ESD	-	86/2676 (3.2%)	4/1932 (0.2%)	2229/2387 (93.4%)	1864/2032 (91.7%)
	-	MD −49.86 (−71.62 to −28.10)	OR 0.37 (0.24–0.57)	OR 14.94 (7.26–30.74)	OR 0.10 (0.09–0.13)	OR 0.14 (0.12–0.17)

MD: mean difference; ND: no data; OR: odds ratio; SMD: standard mean difference; WMD: weighted mean difference. The 95% confidence intervals are shown in parenthesis for MD, OR, SMD, and WMD.

## References

[1] Sung H., Ferlay J., Siegel R.L., Laversanne M., Soerjomataram I., Jemal A., Bray F. (2021). Global Cancer Statistics 2020: GLOBOCAN Estimates of Incidence and Mortality Worldwide for 36 Cancers in 185 Countries. CA Cancer J. Clin..

[2] Stewart B.W., Wild C.P. (2014). World Cancer Report 2014.

[3] World Health Organization (2020). Cancer Tomorrow—International Agency for Research on Cancer.

[4] Weir H.K., Thompson T.D., Stewart S.L., White M.C. (2021). Cancer Incidence Projections in the United States Between 2015 and 2050. Prev. Chronic Dis..

[5] Correa P., Piazuelo M.B. (2012). The Gastric Precancerous Cascade. J. Dig. Dis..

[6] Hamashima C., Kato K., Miyashiro I., Nishida H., Takaku R., Terasawa T., Yoshikawa T., Honjo S., Inoue K., Nakayama T. (2018). Update Version of the Japanese Guidelines for Gastric Cancer Screening. Jpn. J. Clin. Oncol..

[7] Park H.A., Nam S.Y., Lee S.K., Kim S.G., Shim K.N., Park S.M., Lee S.Y., Han H.S., Shin Y.M., Kim K.M. (2015). The Korean Guideline for Gastric Cancer Screening. J. Korean Med. Assoc..

[8] Japanese Gastric Cancer Association (1998). Japanese Classification of Gastric Carcinoma—2nd English Edition. Gastric Cancer.

[9] Yanzhang W., Guanghua L., Zhihao Z., Zhixiong W., Zhao W. (2021). The Risk of Lymph Node Metastasis in Gastric Cancer Conforming to Indications of Endoscopic Resection and Pylorus-Preserving Gastrectomy: A Single-Center Retrospective Study. BMC Cancer.

[10] Park Y.M., Cho E., Kang H.Y., Kim J.M. (2011). The Effectiveness and Safety of Endoscopic Submucosal Dissection Compared with Endoscopic Mucosal Resection for Early Gastric Cancer: A Systematic Review and Metaanalysis. Surg. Endosc..

[11] Suzuki H., Takizawa K., Hirasawa T., Takeuchi Y., Ishido K., Hoteya S., Yano T., Tanaka S., Endo M., Nakagawa M. (2019). Short-Term Outcomes of Multicenter Prospective Cohort Study of Gastric Endoscopic Resection: ‘Real-World Evidence’ in Japan. Dig. Endosc..

[12] Kim S.G., Park C.M., Lee N.R., Kim J., Lyu D.H., Park S.H., Choi I.J., Lee W.S., Park S.J., Kim J.J. (2018). Long-Term Clinical Outcomes of Endoscopic Submucosal Dissection in Patients with Early Gastric Cancer: A Prospective Multicenter Cohort Study. Gut Liver.

[13] Endoscopic Classification Review Group (2005). Update on the Paris Classification of Superficial Neoplastic Lesions in the Digestive Tract. Endoscopy.

[14] Dixon M.F. (2002). Gastrointestinal Epithelial Neoplasia: Vienna Revisited. Gut.

[15] Ribeiro H., Libânio D., Castro R., Ferreira A., Barreiro P., Boal Carvalho P., Capela T., Pimentel-Nunes P., Santos C., Dinis-Ribeiro M. (2019). Reliability of Paris Classification for Superficial Neoplastic Gastric Lesions Improves with Training and Narrow Band Imaging. Endosc. Int. Open.

[16] Gotoda T., Yanagisawa A., Sasako M., Ono H., Nakanishi Y., Shimoda T., Kato Y. (2000). Incidence of Lymph Node Metastasis from Early Gastric Cancer: Estimation with a Large Number of Cases at Two Large Centers. Gastric Cancer.

[17] Nakahara K., Tsuruta O., Tateishi H., Arima N., Takeda J., Toyonaga A., Sata M. (2004). Extended Indication Criteria for Endoscopic Mucosal Resection of Early Gastric Cancer with Special Reference to Lymph Node Metastasis Examination by Multivariate Analysis. Kurume Med. J..

[18] Hirasawa T., Gotoda T., Miyata S., Kato Y., Shimoda T., Taniguchi H., Fujisaki J., Sano T., Yamaguchi T. (2009). Incidence of Lymph Node Metastasis and the Feasibility of Endoscopic Resection for Undifferentiated-Type Early Gastric Cancer. Gastric Cancer.

[19] Chen J., Zhao G., Wang Y. (2020). Analysis of Lymph Node Metastasis in Early Gastric Cancer: A Single Institutional Experience from China. World J. Surg. Oncol..

[20] Hasuike N., Ono H., Boku N., Mizusawa J., Takizawa K., Fukuda H., Oda I., Doyama H., Kaneko K., Hori S. (2017). A Non-Randomized Confirmatory Trial of an Expanded Indication for Endoscopic Submucosal Dissection for Intestinal-Type Gastric Cancer (CT1a): The Japan Clinical Oncology Group Study (JCOG0607). Gastric Cancer.

[21] Takizawa K., Ono H., Hasuike N., Takashima A., Minashi K., Boku N., Kushima R., Katayama H., Ogawa G., Fukuda H. (2021). A Nonrandomized, Single-Arm Confirmatory Trial of Expanded Endoscopic Submucosal Dissection Indication for Undifferentiated Early Gastric Cancer: Japan Clinical Oncology Group Study (JCOG1009/1010). Gastric Cancer.

[22] Ono H., Yao K., Fujishiro M., Oda I., Uedo N., Nimura S., Yahagi N., Iishi H., Oka M., Ajioka Y. (2021). Guidelines for Endoscopic Submucosal Dissection and Endoscopic Mucosal Resection for Early Gastric Cancer (Second Edition). Dig. Endosc..

[23] Pimentel-Nunes P., Libânio D., Bastiaansen B.A.J., Bhandari P., Bisschops R., Bourke M.J., Esposito G., Lemmers A., Maselli R., Messmann H. (2022). Endoscopic Submucosal Dissection for Superficial Gastrointestinal Lesions: European Society of Gastrointestinal Endoscopy (ESGE) Guideline—Update 2022. Endoscopy.

[24] Japanese Gastric Cancer Association (2023). Japanese Gastric Cancer Treatment Guidelines 2021 (6th Edition). Gastric Cancer.

[25] Figueirôa G., Pimentel-Nunes P., Dinis-Ribeiro M., Libânio D. (2019). Gastric Endoscopic Submucosal Dissection: A Systematic Review and Meta-Analysis on Risk Factors for Poor Short-Term Outcomes. Eur. J. Gastroenterol. Hepatol..

[26] Kim E.H., Park J.C., Song I.J., Kim Y.J., Joh D.H., Hahn K.Y., Lee Y.K., Kim H.Y., Chung H., Shin S.K. (2016). Prediction Model for Non-Curative Resection of Endoscopic Submucosal Dissection in Patients with Early Gastric Cancer. Gastrointest. Endosc..

[27] Nam H.S., Choi C.W., Kim S.J., Kang D.H., Kim H.W., Park S.B., Ryu D.G., Choi J.S. (2018). Preprocedural Prediction of Non-Curative Endoscopic Submucosal Dissection for Early Gastric Cancer. PLoS ONE.

[28] Horiuchi Y., Fujisaki J., Yamamoto N., Ishizuka N., Omae M., Ishiyama A., Yoshio T., Hirasawa T., Yamamoto Y., Nagahama M. (2018). Undifferentiated-Type Component Mixed with Differentiated-Type Early Gastric Cancer Is a Significant Risk Factor for Endoscopic Non-Curative Resection. Dig. Endosc..

[29] Xu P., Wang Y., Dang Y., Huang Q., Wang J., Zhang W., Zhang Y., Zhang G. (2020). Predictive Factors and Long-Term Outcomes of Early Gastric Carcinomas in Patients with Non-Curative Resection by Endoscopic Submucosal Dissection. Cancer Manag. Res..

[30] Ma X., Zhang Q., Zhu S., Zhang S., Sun X. (2021). Risk Factors and Prediction Model for Non-Curative Resection of Early Gastric Cancer With Endoscopic Resection and the Evaluation. Front. Med..

[31] Kadota T., Hasuike N., Ono H., Boku N., Mizusawa J., Oda I., Oyama T., Horiuchi Y., Hirasawa K., Yoshio T. (2022). Clinical Factors Associated with Noncurative Endoscopic Submucosal Dissection for the Expanded Indication of Intestinal-type Early Gastric Cancer: Post Hoc Analysis of a Multi-institutional, Single-arm, Confirmatory Trial (JCOG0607). Dig. Endosc..

[32] Yang P., Zheng X.-D., Wang J.-M., Geng W.-B., Wang X. (2022). Undifferentiated-Predominant Mixed-Type Early Gastric Cancer Is More Aggressive than Pure Undifferentiated Type: A Systematic Review and Meta-Analysis. BMJ Open.

[33] Du M.Z., Gan W.J., Yu J., Liu W., Zhan S.H., Huang S., Huang R.P., Chuan Guo L., Huang Q. (2018). Risk Factors of Lymph Node Metastasis in 734 Early Gastric Carcinoma Radical Resections in a Chinese Population: Nodes Metastasis in Early Gastric Cancer. J. Dig. Dis..

[34] Milhomem L.M., Milhomem-Cardoso D.M., da Mota O.M., Mota E.D., Kagan A., Filho J.B.S. (2021). Risk of Lymph Node Metastasis in Early Gastric Cancer and Indications for Endoscopic Resection: Is It Worth Applying the East Rules to the West?. Surg. Endosc..

[35] Oh Y.J., Kim D.H., Han W.H., Eom B.W., Kim Y.I., Yoon H.M., Lee J.Y., Kim C.G., Kook M.-C., Choi I.J. (2021). Risk Factors for Lymph Node Metastasis in Early Gastric Cancer without Lymphatic Invasion after Endoscopic Submucosal Dissection. Eur. J. Surg. Oncol..

[36] Abe S., Oda I., Shimazu T., Kinjo T., Tada K., Sakamoto T., Kusano C., Gotoda T. (2011). Depth-Predicting Score for Differentiated Early Gastric Cancer. Gastric Cancer.

[37] Nakayoshi T., Tajiri H., Matsuda K., Kaise M., Ikegami M., Sasaki H. (2004). Magnifying Endoscopy Combined with Narrow Band Imaging System for Early Gastric Cancer: Correlation of Vascular Pattern with Histo-pathology (Including Video). Endoscopy.

[38] Yokoyama A., Inoue H., Minami H., Wada Y., Sato Y., Satodate H., Hamatani S., Kudo S. (2010). Novel Nar-row-Band Imaging Magnifying Endoscopic Classification for Early Gastric Cancer. Dig. Liver Dis..

[39] Tanaka K., Toyoda H., Kadowaki S., Kosaka R., Shiraishi T., Imoto I., Shiku H., Adachi Y. (2006). Features of Early Gastric Cancer and Gastric Adenoma by Enhanced-Magnification Endoscopy. J. Gastroenterol..

[40] Ok K.-S., Kim G.H., Park D.Y., Lee H.J., Jeon H.K., Baek D.H., Lee B.E., Song G.A. (2016). Magnifying En-doscopy with Narrow Band Imaging of Early Gastric Cancer: Correlation with Histopathology and Mucin Phenotype. Gut Liver.

[41] Kanesaka T., Uedo N., Doyama H., Yoshida N., Nagahama T., Ohtsu K., Uchita K., Kojima K., Ueo T., Takahashi H. (2021). Diagnosis of Histological Type of Early Gastric Cancer by Magnifying Narrow-band Imaging: A Multicenter Prospective Study. DEN Open.

[42] Inoue H., Takeshita K., Hori H., Muraoka Y., Yoneshima H., Endo M. (1993). Endoscopic Mucosal Resection with a Cap-Fitted Panendoscope for Esophagus, Stomach, and Colon Mucosal Lesions. Gastrointest. Endosc..

[43] Ono H., Kondo H., Gotoda T., Shirao K., Yamaguchi H., Saito D., Hosokawa K., Shimoda T., Yoshida S. (2001). Endoscopic Mucosal Resection for Treatment of Early Gastric Cancer. Gut.

[44] Gotoda T., Kondo H., Ono H., Saito Y., Yamaguchi H., Saito D., Yokota T. (1999). A New Endoscopic Mucosal Resection Procedure Using an Insulation-Tipped Electrosurgical Knife for Rectal Flat Lesions: Report of Two Cases. Gastrointest. Endosc..

[45] Tao M., Zhou X., Hu M., Pan J. (2019). Endoscopic Submucosal Dissection versus Endoscopic Mucosal Resection for Patients with Early Gastric Cancer: A Meta-Analysis. BMJ Open.

[46] Lian J., Chen S., Zhang Y., Qiu F. (2012). A Meta-Analysis of Endoscopic Submucosal Dissection and EMR for Early Gastric Cancer. Gastrointest. Endosc..

[47] Facciorusso A., Antonino M., Di Maso M., Muscatiello N. (2014). Endoscopic Submucosal Dissection vs Endoscopic Mucosal Resection for Early Gastric Cancer: A Meta-Analysis. World J. Gastrointest. Endosc..

[48] Zhao Y., Wang C. (2018). Long-Term Clinical Efficacy and Perioperative Safety of Endoscopic Submucosal Dissection versus Endoscopic Mucosal Resection for Early Gastric Cancer: An Updated Meta-Analysis. BioMed Res. Int..

[49] Tanabe S., Ishido K., Matsumoto T., Kosaka T., Oda I., Suzuki H., Fujisaki J., Ono H., Kawata N., Oyama T. (2017). Long-Term Outcomes of Endoscopic Submucosal Dissection for Early Gastric Cancer: A Multicenter Collaborative Study. Gastric Cancer.

[50] Peng L.J., Tian S.N., Lu L., Chen H., Ouyang Y.Y., Wu Y.J. (2015). Outcome of Endoscopic Submucosal Dissection for Early Gastric Cancer of Conventional and Expanded Indications: Systematic Review and Meta-Analysis. J. Dig. Dis..

[51] Suzuki H., Ono H., Hirasawa T., Takeuchi Y., Ishido K., Hoteya S., Yano T., Tanaka S., Toya Y., Nakagawa M. (2022). Long-Term Survival After Endoscopic Resection For Gastric Cancer: Real-World Evidence From a Multicenter Prospective Cohort. Clin. Gastroenterol. Hepatol..

[52] Shichijo S., Uedo N., Kanesaka T., Ohta T., Nakagawa K., Shimamoto Y., Ohmori M., Arao M., Iwatsubo T., Suzuki S. (2020). Long-term Outcomes after Endoscopic Submucosal Dissection for Differentiated-type Early Gastric Cancer That Fulfilled Expanded Indication Criteria: A Prospective Cohort Study. J. Gastroenterol. Hepatol..

[53] Libânio D., Costa M.N., Pimentel-Nunes P., Dinis-Ribeiro M. (2016). Risk Factors for Bleeding after Gastric Endoscopic Submucosal Dissection: A Systematic Review and Meta-Analysis. Gastrointest. Endosc..

[54] Uedo N., Takeuchi Y., Yamada T., Ishihara R., Ogiyama H., Yamamoto S., Kato M., Tatsumi K., Masuda E., Tamai C. (2007). Effect of a Proton Pump Inhibitor or an H2-Receptor Antagonist on Prevention of Bleeding From Ulcer After Endoscopic Submucosal Dissection of Early Gastric Cancer: A Prospective Randomized Controlled Trial. Am. J. Gastroenterol..

[55] Yang Z., Wu Q., Liu Z., Wu K., Fan D. (2011). Proton Pump Inhibitors versus Histamine-2-Receptor Antagonists for the Management of Iatrogenic Gastric Ulcer after Endoscopic Mucosal Resection or Endoscopic Submucosal Dissection: A Meta-Analysis of Randomized Trials. Digestion.

[56] Nishizawa T., Suzuki H., Akimoto T., Maehata T., Morizane T., Kanai T., Yahagi N. (2016). Effects of Preoperative Proton Pump Inhibitor Administration on Bleeding after Gastric Endoscopic Submucosal Dissection: A Systematic Review and Meta-analysis. United Eur. Gastroenterol. J..

[57] Takizawa K., Oda I., Gotoda T., Yokoi C., Matsuda T., Saito Y., Saito D., Ono H. (2008). Routine Coagulation of Visible Vessels May Prevent Delayed Bleeding after Endoscopic Submucosal Dissection—An Analysis of Risk Factors. Endoscopy.

[58] Chen Y., Zhao X., Wang D., Liu X., Chen J., Song J., Bai T., Hou X. (2022). Endoscopic Delivery of Polymers Reduces Delayed Bleeding after Gastric Endoscopic Submucosal Dissection: A Systematic Review and Meta-Analysis. Polymers.

[59] Ding X., Luo H., Duan H. (2019). Risk Factors for Perforation of Gastric Endoscopic Submucosal Dissection: A Systematic Review and Meta-Analysis. Eur. J. Gastroenterol. Hepatol..

[60] Zhao J., Sun Z., Liang J., Guo S., Huang D. (2022). Endoscopic Submucosal Dissection for Early Gastric Cancer in Elderly vs. Non-Elderly Patients: A Systematic Review and Meta-Analysis. Front. Oncol..

[61] Abdelfatah M.M., Barakat M., Ahmad D., Ibrahim M., Ahmed Y., Kurdi Y., Grimm I.S., Othman M.O. (2019). Long-term Outcomes of Endoscopic Submucosal Dissection versus Surgery in Early Gastric Cancer: A Systematic Review and Meta-Analysis. Eur. J. Gastroenterol. Hepatol..

[62] Gu L., Khadaroo P.A., Chen L., Li X., Zhu H., Zhong X., Pan J., Chen M. (2019). Comparison of Long-Term Outcomes of Endoscopic Submucosal Dissection and Surgery for Early Gastric Cancer: A Systematic Review and Meta-Analysis. J. Gastrointest. Surg..

[63] Li H., Feng L.-Q., Bian Y.-Y., Yang L.-L., Liu D.-X., Huo Z.-B., Zeng L. (2019). Comparison of Endoscopic Submucosal Dissection with Surgical Gastrectomy for Early Gastric Cancer: An Updated Meta-Analysis. World J. Gastrointest. Oncol..

[64] Liu Q., Ding L., Qiu X., Meng F. (2019). Updated Evaluation of Endoscopic Submucosal Dissection versus Surgery for Early Gastric Cancer: A Systematic Review and Meta-Analysis. Int. J. Surg..

[65] Xu X., Zheng G., Gao N., Zheng Z. (2022). Long-Term Outcomes and Clinical Safety of Expanded Indication Early Gastric Cancer Treated with Endoscopic Submucosal Dissection versus Surgical Resection: A Meta-Analysis. BMJ Open.

[66] Nakamura R., Omori T., Mayanagi S., Irino T., Wada N., Kawakubo H., Kameyama K., Kitagawa Y. (2019). Risk of Lymph Node Metastasis in Undifferentiated-Type Mucosal Gastric Carcinoma. World J. Surg. Oncol..

[67] Huh C.-W., Ma D.W., Kim B.-W., Kim J.S., Lee S.J. (2021). Endoscopic Submucosal Dissection versus Surgery for Undifferentiated-Type Early Gastric Cancer: A Systematic Review and Meta-Analysis. Clin. Endosc..

[68] Yang H.-J., Kim J.-H., Kim N.W., Choi I.J. (2022). Comparison of Long-Term Outcomes of Endoscopic Submucosal Dissection and Surgery for Undifferentiated-Type Early Gastric Cancer Meeting the Expanded Criteria: A Systematic Review and Meta-Analysis. Surg. Endosc..

[69] Kim Y., Kim Y.-W., Choi I.J., Cho J.Y., Kim J.H., Kwon J.-W., Lee J.Y., Lee N.R., Seol S.-Y. (2015). Cost Comparison between Surgical Treatments and Endoscopic Submucosal Dissection in Patients with Early Gastric Cancer in Korea. Gut Liver.

[70] Shin D.W., Hwang H.Y., Jeon S.W. (2017). Comparison of Endoscopic Submucosal Dissection and Surgery for Differentiated Type Early Gastric Cancer within the Expanded Criteria. Clin. Endosc..

[71] Qian M., Sheng Y., Wu M., Wang S., Zhang K. (2022). Comparison between Endoscopic Submucosal Dissection and Surgery in Patients with Early Gastric Cancer. Cancers.

[72] Song W., Qiao X., Gao X. (2015). A Comparison of Endoscopic Submucosal Dissection (ESD) and Radical Surgery for Early Gastric Cancer: A Retrospective Study. World J. Surg. Oncol..

[73] Libânio D., Braga V., Ferraz S., Castro R., Lage J., Pita I., Ribeiro C., Abreu De Sousa J., Dinis-Ribeiro M., Pimentel-Nunes P. (2019). Prospective Comparative Study of Endoscopic Submucosal Dissection and Gastrectomy for Early Neoplastic Lesions Including Patients’ Perspectives. Endoscopy.

[74] Kim Y.-I., Kim Y.A., Kim C.G., Ryu K.W., Kim Y.-W., Sim J.A., Yun Y.H., Choi I.J. (2018). Serial Intermediate-Term Quality of Life Comparison after Endoscopic Submucosal Dissection versus Surgery in Early Gastric Cancer Patients. Surg. Endosc..

[75] Libânio D., Ortigão R., Pimentel-Nunes P., Dinis-Ribeiro M. (2022). Improving the Diagnosis and Treatment of Early Gastric Cancer in the West. GE Port J. Gastroenterol..

[76] Libânio D., Pimentel-Nunes P., Afonso L.P., Henrique R., Dinis-Ribeiro M. (2016). Long-Term Outcomes of Gastric Endoscopic Submucosal Dissection: Focus on Metachronous and Non-Curative Resection Management. GE Port. J. Gastroenterol..

[77] Kawata N., Kakushima N., Takizawa K., Tanaka M., Makuuchi R., Tokunaga M., Tanizawa Y., Bando E., Kawamura T., Sugino T. (2016). Risk Factors for Lymph Node Metastasis and Long-Term Outcomes of Patients with Early Gastric Cancer after Non-Curative Endoscopic Submucosal Dissection. Surg. Endosc..

[78] Hatta W., Gotoda T., Oyama T., Kawata N., Takahashi A., Yoshifuku Y., Hoteya S., Nakagawa M., Hirano M., Esaki M. (2017). A Scoring System to Stratify Curability after Endoscopic Submucosal Dissection for Early Gastric Cancer: “ECura System”. Am. J. Gastroenterol..

[79] Ortigão R., Figueirôa G., Frazzoni L., Pimentel-Nunes P., Hassan C., Dinis-Ribeiro M., Fuccio L., Libânio D. (2022). Risk Factors for Gastric Metachronous Lesions after Endoscopic or Surgical Resection: A Systematic Review and Meta-Analysis. Endoscopy.

[80] Hahn K.Y., Park J.C., Kim E.H., Shin S., Park C.H., Chung H., Shin S.K., Lee S.K., Lee Y.C. (2016). Incidence and Impact of Scheduled Endoscopic Surveillance on Recurrence after Curative Endoscopic Resection for Early Gastric Cancer. Gastrointest. Endosc..

[81] Fan F., Wang Z., Li B., Zhang H. (2020). Effects of Eradicating Helicobacter Pylori on Metachronous Gastric Cancer Prevention: A Systematic Review and Meta-analysis. J. Eval. Clin. Pract..

[82] Bang C.S., Baik G.H., Shin I.S., Kim J.B., Suk K.T., Yoon J.H., Kim Y.S., Kim D.J. (2015). Helicobacter Pylori Eradication for Prevention of Metachronous Recurrence after Endoscopic Resection of Early Gastric Cancer. J. Korean Med. Sci..

[83] Xiao S., Li S., Zhou L., Jiang W., Liu J. (2019). Helicobacter Pylori Status and Risks of Metachronous Recurrence after Endoscopic Resection of Early Gastric Cancer: A Systematic Review and Meta-Analysis. J. Gastroenterol..

[84] Yoon S.B., Park J.M., Lim C.-H., Cho Y.K., Choi M.-G. (2014). Effect of Helicobacter Pylori Eradication on Metachronous Gastric Cancer after Endoscopic Resection of Gastric Tumors: A Meta-Analysis. Helicobacter.

[85] Khan M.Y., Aslam A., Mihali A.B., Shabbir Rawala M., Dirweesh A., Khan S., Adler D.G., Siddiqui A. (2020). Effectiveness of Helicobacter Pylori Eradication in Preventing Metachronous Gastric Cancer and Preneoplastic Lesions. A Systematic Review and Meta-Analysis. Eur. J. Gastroenterol. Hepatol..

[86] Zhao B., Zhang J., Mei D., Luo R., Lu H., Xu H., Huang B. (2020). Does Helicobacter Pylori Eradication Reduce the Incidence of Metachronous Gastric Cancer After Curative Endoscopic Resection of Early Gastric Cancer. J. Clin. Gastroenterol..

[87] Pimentel-Nunes P., Libânio D., Marcos-Pinto R., Areia M., Leja M., Esposito G., Garrido M., Kikuste I., Megraud F., Matysiak-Budnik T. (2019). Management of Epithelial Precancerous Conditions and Lesions in the Stomach (MAPS II): European Society of Gastrointestinal Endoscopy (ESGE), European Helicobacter and Microbiota Study Group (EHMSG), European Society of Pathology (ESP), and Sociedade Portuguesa de Endoscopia Digestiva (SPED) Guideline Update 2019. Endoscopy.

[88] Kobayashi M., Narisawa R., Sato Y., Takeuchi M., Aoyagi Y. (2010). Self-Limiting Risk of Metachronous Gastric Cancers after Endoscopic Resection. Dig. Endosc..

[89] Zhu Y., Wang Q.-C., Xu M.-D., Zhang Z., Cheng J., Zhong Y.-S., Zhang Y.-Q., Chen W.-F., Yao L.-Q., Zhou P.-H. (2019). Application of Convolutional Neural Network in the Diagnosis of the Invasion Depth of Gastric Cancer Based on Conventional Endoscopy. Gastrointest. Endosc..

[90] Tang D., Zhou J., Wang L., Ni M., Chen M., Hassan S., Luo R., Chen X., He X., Zhang L. (2021). A Novel Model Based on Deep Convolutional Neural Network Improves Diagnostic Accuracy of Intramucosal Gastric Cancer (With Video). Front. Oncol..

[91] Yoon H.J., Kim S., Kim J.-H., Keum J.-S., Oh S.-I., Jo J., Chun J., Youn Y.H., Park H., Kwon I.G. (2019). A Lesion-Based Convolutional Neural Network Improves Endoscopic Detection and Depth Prediction of Early Gastric Cancer. J. Clin. Med..

[92] Nagao S., Tsuji Y., Sakaguchi Y., Takahashi Y., Minatsuki C., Niimi K., Yamashita H., Yamamichi N., Seto Y., Tada T. (2020). Highly Accurate Artificial Intelligence Systems to Predict the Invasion Depth of Gastric Cancer: Efficacy of Conventional White-Light Imaging, Nonmagnifying Narrow-Band Imaging, and Indigo-Carmine Dye Contrast Imaging. Gastrointest. Endosc..

[93] Wu L., Wang J., He X., Zhu Y., Jiang X., Chen Y., Wang Y., Huang L., Shang R., Dong Z. (2022). Deep Learning System Compared with Expert Endoscopists in Predicting Early Gastric Cancer and Its Invasion Depth and Differentiation Status (with Videos). Gastrointest. Endosc..

[94] Hamada K., Kawahara Y., Tanimoto T., Ohto A., Toda A., Aida T., Yamasaki Y., Gotoda T., Ogawa T., Abe M. (2021). Application of Convolutional Neural Networks for Evaluating the Depth of Invasion of Early Gastric Cancer Based on Endoscopic Images. J. Gastroenterol. Hepatol..

[95] Jiang K., Jiang X., Pan J., Wen Y., Huang Y., Weng S., Lan S., Nie K., Zheng Z., Ji S. (2021). Current Evidence and Future Perspective of Accuracy of Artificial Intelligence Application for Early Gastric Cancer Diagnosis With Endoscopy: A Systematic and Meta-Analysis. Front. Med. (Lausanne).

[96] Xie F., Zhang K., Li F., Ma G., Ni Y., Zhang W., Wang J., Li Y. (2021). Diagnostic Accuracy of Convolutional Neural Network–Based Endoscopic Image Analysis in Diagnosing Gastric Cancer and Predicting Its Invasion Depth: A Systematic Review and Meta-Analysis. Gastrointest. Endosc..

[97] Kim J.-H., Oh S.-I., Han S.-Y., Keum J.-S., Kim K.-N., Chun J.-Y., Youn Y.-H., Park H. (2022). An Optimal Artificial Intelligence System for Real-Time Endoscopic Prediction of Invasion Depth in Early Gastric Cancer. Cancers.

[98] Goto A., Kubota N., Nishikawa J., Ogawa R., Hamabe K., Hashimoto S., Ogihara H., Hamamoto Y., Yanai H., Miura O. (2023). Cooperation between Artificial Intelligence and Endoscopists for Diagnosing Invasion Depth of Early Gastric Cancer. Gastric Cancer.

[99] Abe N., Mori T., Takeuchi H., Yoshida T., Ohki A., Ueki H., Yanagida O., Masaki T., Sugiyama M., Atomi Y. (2005). Laparoscopic Lymph Node Dissection after Endoscopic Submucosal Dissection: A Novel and Minimally Invasive Approach to Treating Early-Stage Gastric Cancer. Am. J. Surg..

[100] Abe N., Takeuchi H., Ohki A., Yanagida O., Masaki T., Mori T., Sugiyama M. (2011). Long-Term Outcomes of Combination of Endoscopic Submucosal Dissection and Laparoscopic Lymph Node Dissection without Gastrectomy for Early Gastric Cancer Patients Who Have a Potential Risk of Lymph Node Metastasis. Gastrointest. Endosc..

[101] Wang Z., Dong Z.-Y., Chen J.-Q., Liu J.-L. (2011). Diagnostic Value of Sentinel Lymph Node Biopsy in Gastric Cancer: A Meta-Analysis. Ann. Surg. Oncol..

[102] Huang L., Wei T., Chen J., Zhou D. (2017). Feasibility and Diagnostic Performance of Dual-Tracer-Guided Sentinel Lymph Node Biopsy in CT1-2N0M0 Gastric Cancer: A Systematic Review and Meta-Analysis of Diagnostic Studies. World J. Surg. Oncol..

[103] Skubleny D., Dang J.T., Skulsky S., Switzer N., Tian C., Shi X., de Gara C., Birch D.W., Karmali S. (2018). Diagnostic Evaluation of Sentinel Lymph Node Biopsy Using Indocyanine Green and Infrared or Fluorescent Imaging in Gastric Cancer: A Systematic Review and Meta-Analysis. Surg. Endosc..

[104] Huang Y., Pan M., Deng Z., Ji Y., Chen B. (2021). How Useful Is Sentinel Lymph Node Biopsy for the Status of Lymph Node Metastasis in CT1N0M0 Gastric Cancer? A Systematic Review and Meta-Analysis. Updates Surg..

[105] Huang Y., Pan M., Chen B. (2021). A Systematic Review and Meta-Analysis of Sentinel Lymph Node Biopsy in Gastric Cancer, an Optimization of Imaging Protocol for Tracer Mapping. World J. Surg..

[106] Zhong Q., Chen Q.-Y., Huang X.-B., Lin G.-T., Liu Z.-Y., Chen J.-Y., Wang H.-G., Weng K., Li P., Xie J.-W. (2021). Clinical Implications of Indocyanine Green Fluorescence Imaging-Guided Laparoscopic Lymphadenectomy for Patients with Gastric Cancer: A Cohort Study from Two Randomized, Controlled Trials Using Individual Patient Data. Int. J. Surg..

[107] Park D.J., Park Y.S., Son S.Y., Lee J.-H., Lee H.S., Park Y.S., Lee K.H., Kim Y.H., Park K.U., Lee W.W. (2018). Long-Term Oncologic Outcomes of Laparoscopic Sentinel Node Navigation Surgery in Early Gastric Cancer: A Single-Center, Single-Arm, Phase II Trial. Ann. Surg. Oncol..

